# Social defeat stress-specific increase in c-Fos expression in the extended amygdala in mice: Involvement of dopamine D1 receptor in the medial prefrontal cortex

**DOI:** 10.1038/s41598-019-52997-7

**Published:** 2019-11-13

**Authors:** Chisato Numa, Hirotaka Nagai, Masayuki Taniguchi, Midori Nagai, Ryota Shinohara, Tomoyuki Furuyashiki

**Affiliations:** 1 Division of Pharmacology, Graduate School of Medicine, Kobe University, Kobe, 650-0017 Japan; 2 Japan Agency for Medical Research and Development, Tokyo, 100-0004 Japan; 3 Present Address: Department of Psychiatry, Yale University School of Medicine, New Haven, CT 06519 USA

**Keywords:** Neural circuits, Social behaviour, Stress and resilience

## Abstract

We recently reported that dopamine D1 receptor in the medial prefrontal cortex (mPFC) is activated by subthreshold social defeat stress and suppresses the induction of depressive-like behavior in mice. However, which mPFC projection(s) mediates this antidepressant-like effect remains poorly understood. Here we show that social defeat stress specifically increased c-Fos expression, a marker for neuronal activity, in distinct brain regions involved in emotional regulation, relative to novelty-induced exploration. Among these brain areas, D1 knockdown in the mPFC decreased social defeat stress-induced c-Fos expression in the interstitial nucleus of the posterior limb of the anterior commissure (IPAC), a subregion of the extended amygdala. Using retrograde adeno-associated virus vectors and transgenic mice expressing Cre recombinase under the D1 promoter, we also found that D1-expressing deep-layer pyramidal neurons in the mPFC send direct projections to the IPAC. These findings indicate that social defeat stress specifically activates neurons in distinct brain areas, among which the IPAC is regulated by dopamine D1 receptor in the mPFC perhaps through direct projections. Thus, this study provides hints toward identifying neural circuits that underlie antidepressant-like effects of stress-induced dopamine D1 receptor signaling in the mPFC.

## Introduction

Stress caused by aversive and demanding conditions induces different biological responses, depending on the stress conditions. It is thought that brief and moderate stress evokes adaptive responses, such as “fight-or-flight response”, and promotes habituation and resilience to stress. By contrast, prolonged or excessive stress may induce cognitive and affective dysfunctions, and predisposes to mental disorders^1,2^. Previous studies using rodent stress models including restraint stress, chronic unpredictable stress and social defeat stress have revealed that stress alters structures and activities of neurons in several brain areas including the medial prefrontal cortex (mPFC), hippocampus, and nucleus accumbens (NAc)^3–7^.

It has been shown that repeated social defeat stress decreases firing rates of dopamine neurons in the ventral tegmental area (VTA) projecting to the mPFC^8^ and stress-induced dopaminergic response in the mPFC^9^. These dopaminergic deficits have been shown to be crucial for repeated social defeat stress-induced social avoidance. On the other hand, we recently reported that subthreshold stress induced by single exposure to social defeat stress induces dopamine D1 receptor signaling in the mPFC^10^. Knockdown of dopamine D1 receptor in excitatory neurons in the mPFC facilitated the induction of social avoidance after single or repeated exposure to social defeat stress. Consistently, it was reported that dopamine D1 receptor in the mPFC is crucial for antidepressant-like effects of ketamine, and that infusion of a D1-like receptor agonist induces antidepressant-like effects^11^. These findings indicate that dopamine D1 receptor signaling induced by single exposure to social defeat stress exerts antidepressant-like effects.

Previous studies using optogenetic manipulations have revealed roles of mPFC projections to several brain areas, such as NAc, basolateral amygdala (BLA), lateral habenula, and dorsal raphe nucleus (DRN), in regulating depressive-like and anxiety-like behaviors^12^. Stimulation of these projections, except that to the lateral habenula, appears to reduce depressive-like and/or anxiety-like behaviors. Besides these projections, mPFC pyramidal neurons send direct and indirect projections to various other brain areas, many of which have been suggested to be involved in stress responses^13^. However, previous studies have not systematically examined which mPFC projections are regulated by dopamine D1 receptor signaling. Thus, which mPFC projection(s) mediates the antidepressant-like effects of stress-induced dopamine D1 receptor signaling remains poorly understood.

In the present study, using c-Fos expression, a marker for neuronal activity, we found that single exposure to social defeat stress specifically activates distinct brain areas including the extended amygdala, relative to novelty-induced exploration. Among these brain areas, knockdown of dopamine D1 receptor in the mPFC decreased social defeat stress-induced c-Fos expression in the interstitial nucleus of the posterior limb of the anterior commissure (IPAC), a subregion of the extended amygdala.

## Results

### Social defeat stress specifically induces c-Fos expression in distinct brain areas

**Figure 1:**
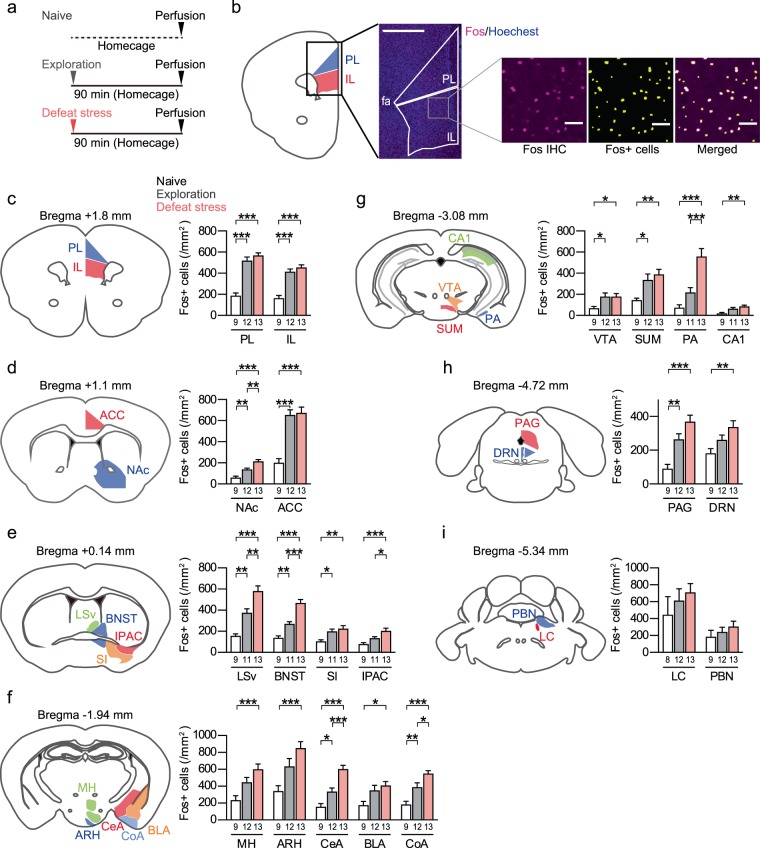
Social defeat stress specifically increased c-Fos expression in distinct brain areas, relative to novelty-induced exploration.(**a**) A behavioral schedule. Mice received social defeat stress for 10 min (the defeat stress group) or were allowed to explore a novel cage for 10 min (the exploration group), and were kept undisturbed in their homecages for 90 min before being sacrificed for immunohistochemistry. As the naïve group, mice were left undisturbed in their homecages until sacrifice. (**b**) Automatic detection and counting of c-Fos-positive cells (Fos + cells) visualized by immunohistochemistry (Fos IHC). For example, the prelimbic cortex (PL) and the infralimbic cortex (IL) adjacent to anterior forceps (fa) were defined in a fluorescent image of c-Fos immunoreactivity (magenta) and nuclear staining with Hoechst33342 (blue), based on corresponding brain areas in the Allen Mouse Brain Atlas, as shown in the left. A magnified image of c-Fos immunoreactivity (magenta) corresponding to the inset in the fluorescent image and binarized signals of automatically designated Fos + cells (yellow) are shown in the right. Note that c-Fos signals are well overlapped by the binarized Fos + cells. Scale bars: 500 μm (left) and 50 μm (right). (**c–i**) Quantification of the numbers of Fos + cells in the naïve group, the exploration group and the defeat stress group. The number of data points for each group is shown below each bar. Schematics were drawn based on the Allen Mouse Brain Atlas. PL; prelimbic cortex, IL; infralimbic cortex, NAc; nucleus accumbens, ACC; anterior cingulate cortex, LSv; lateral septal nucleus ventral part, BNST; bed nucleus of the stria terminalis, SI; substantia innominata, IPAC; interstitial nucleus of the posterior limb of the anterior commissure, MH; medial hypothalamus, ARH; arcuate nucleus, CeA; central amygdala, BLA; basolateral amygdala, CoA; cortical amygdala, VTA; ventral tegmental area, SUM; supramammillary nucleus, PA; posterior amygdala, PAG; periaqueductal gray, DRN; dorsal raphe nucleus, LC; locus coeruleus, PBN; parabrachial nucleus. Values are expressed as means ± SEM. *P < 0.05, **P < 0.01, ***P < 0.001 for multiple comparison tests with Tukey-Kramer correction.

To examine brain regions that are activated by single exposure to social defeat stress, we exposed adult male C57BL/6 N mice to social defeat stress for 10 min (defeat stress group), and subjected these mice to immunohistochemistry for c-Fos as a marker for neuronal activity in several brain areas associated with emotional regulation at 90 min after the stress (Fig. 1a,b). In this experiment, we set two different control groups, the naïve group, in which mice were left undisturbed in their home cages, and the exploration group, in which mice were placed in a novel cage and engaged in novelty-induced exploration for 10 min (Fig. 1a). These two control groups allowed us to identify brain areas which were selectively activated by social defeat stress relative to novelty-induced exploration. In most brain regions tested, one-way ANOVA revealed significant difference among the naïve group, the exploration group and the defeat stress group: prelimbic cortex (PL; *P* < 0.0001, *F*(2, 31) = 47.74), infralimbic cortex (IL; *P* < 0.0001, *F*(2, 31) = 35.08), NAc (*P* < 0.0001, *F*(2, 31) = 24.95), anterior cingulate cortex (ACC; *P* < 0.0001, *F*(2, 31) = 24.95), lateral septal nucleus ventral part (LSv; *P* < 0.0001, *F*(2, 30) = 25.68), bed nucleus of the stria terminalis (BNST; *P* < 0.0001, *F*(2, 30) = 39.17), substantia innominata (SI; *P* = 0.0052, *F*(2, 30) = 6.288), IPAC (*P* < 0.0006, *F*(2, 30) = 9.497), medial hypothalamus (MH; *P* = 0.0008, *F*(2, 31) = 9.038), arcuate nucleus of the hypothalamus (ARH; *P* = 0.0006, *F*(2, 31) = 9.412), central amygdala (CeA; *P* < 0.0001, *F*(2, 31) = 25.74), BLA (*P* = 0.0131, *F*(2, 31) = 5.002), cortical amygdala (CoA; *P* < 0.0001, *F*(2, 31) = 17.37), VTA (*P* = 0.0217, *F*(2, 31) = 4.344), supramammillary nucleus (SUM; *P* = 0.0038, *F*(2, 31) = 6.716), posterior amygdala (PA; *P* < 0.0001, *F*(2, 30) = 18.08), hippocampal CA1 region (*P* = 0.0027, *F*(2, 30) = 7.240), periaqueductal gray (PAG; *P* < 0.0001, *F*(2, 31) = 15.67), and DRN (*P* = 0.0096, *F*(2, 31) = 5.411) (Fig. 1c–h). No significant difference was found in the locus coeruleus (LC; *P* = 0.4786, *F*(2, 30) = 0.7552) and parabrachial nucleus (PBN; *P* = 0.4370, *F*(2, 31) = 0.8503) (Fig. 1i). In the brain areas with significant difference among the three groups, social defeat stress increased the numbers of c-Fos-positive cells, compared with the naïve group: PL (*P* < 0.0001, *q*(31) = 13.10), IL (*P* < 0.0001, *q*(31) = 11.28), NAc (*P* < 0.0001, *q*(31) = 9.930), ACC (*P* < 0.0001, *q*(31) = 9.144), LSv (*P* < 0.0001, *q*(31) = 10.10), BNST (*P* < 0.0001, *q*(30) = 12.22), SI (*P* = 0.0046, *q*(30) = 4.885), IPAC (*P* = 0.0005, *q*(30) = 6.076), MH (*P* = 0.0005, *q*(31) = 6.011), ARH (*P* = 0.0004, *q*(31) = 6.135), CeA (*P* < 0.0001, *q*(31) = 9.895), BLA (*P* = 0.0111, *q*(31) = 4.387), CoA (*P* < 0.0001, *q*(31) = 8.330), VTA (*P* = 0.0337, *q*(31) = 3.729), SUM (*P* = 0.0033, *q*(31) = 5.045), PA (*P* < 0.0001, *q*(30) = 8.041), hippocampal CA1 region (*P* = 0.0019, *q*(30) = 5.366), PAG (*P* < 0.0001, *q*(31) = 7.912) and DRN (*P* = 0.0072, *q*(31) = 4.629) (Fig. 1c–h). Among these brain areas, more c-Fos-positive cells in the defeat group relative to the exploration group were found in NAc (*P* = 0.0018, *q*(31) = 5.375), LSv (*P* = 0.0027, *q*(30) = 5.180), BNST (*P* < 0.0001, *q*(30) = 7.742), IPAC (*P* = 0.0443, *q*(30) = 3.564), CeA (*P* = 0.0002, *q*(31) = 6.397), CoA (*P* = 0.0238, *q*(31) = 3.941) and PA (*P* = 0.0006, *q*(30) = 5.977). In most of these brain areas, novelty-induced exploration increased the number of c-Fos-positive cells relative to the naïve group, albeit to the lesser extent than social defeat stress. These findings indicate that neurons in distinct brain regions including the extended amygdala, an anatomical continuity of GABAergic neurons in BNST, IPAC and CeA involved in emotional regulation^14^, were selectively activated by social defeat stress, whereas neurons in other brain regions including PL, IL, ACC, SI, VTA and SUM were similarly activated by both the exploration and the stress.

### Dopamine D1 receptor in the mPFC regulates social defeat stress-induced c-Fos expression in the IPAC

**Figure 2:**
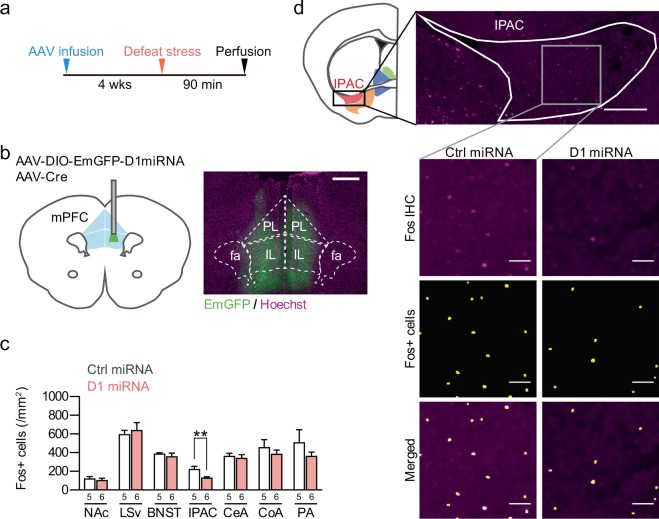
Knockdown of dopamine D1 receptor in the mPFC decreased social defeat stress-induced c-Fos expression in the IPAC. (**a**) An experimental schedule. Mice received bilateral infusions of an AAV vector expressing either artificial microRNA targeting dopamine D1 receptor (AAV-DIO-EmGFP-D1miRNA) or control sequence (AAV-DIO-EmGFP-CtrlmiRNA) with EmGFP in the presence of Cre recombinase. Another AAV vector expressing Cre recombinase under the CMV promoter (AAV-Cre) was simultaneously infused to enable expression of D1 and control miRNA with EmGFP. Four weeks later, they received single exposure to social defeat stress and were sacrificed for immunohistochemistry at 90 min later. (**b**) A representative image (right) of EmGFP expression (green) derived from AAV vectors infused into the mPFC (left) and nuclear counterstaining with Hoechst33342 (magenta). Note that EmGFP expression is centered at the infralimbic cortex (IL), but spread to adjacent areas including the prelimbic cortex (PL). Scale bar: 200 μm. (**c**) Quantification of the numbers of Fos + cells in the defeat stress group with expression of control miRNA (Ctrl miRNA) and D1 miRNA in the mPFC. The number of data points for each group is shown below each bar. Values are expressed as means ± SEM. **P < 0.01 for unpaired *t* test. See the legend of Fig. 1 for abbreviations of the names of brain areas. (**d**) Representative images of c-Fos immunoreactivity (magenta) and binarized Fos + cells (yellow) in the IPAC of the defeated mice with expression of control miRNA or D1 miRNA in the mPFC. Note that the D1 knockdown in the mPFC decreased the number of Fos + cells. Scale bar: 200 μm (upper image) and 50 μm (lower images). Schematics were drawn based on the Allen Mouse Brain Atlas.

Since we previously reported that dopamine D1 receptor signaling in the mPFC upon social defeat stress attenuates the induction of social avoidance^10^, we examined whether dopamine D1 receptor in the mPFC is involved in social defeat stress-induced c-Fos expression described above. For this purpose, we knocked down the dopamine D1 receptor specifically in the mPFC with AAV vectors expressing artificial microRNA targeting D1 mRNA with EmGFP (Fig. 2a,b). Specificity and efficacy of the D1 knockdown has been validated elsewhere^10^. We subjected mice with the D1 knockdown to social defeat stress and performed c-Fos immunohistochemistry. Among the brain areas in which social defeat stress specifically induced c-Fos expression relative to novelty-induced exploration, the D1 knockdown significantly decreased the number of c-Fos-positive cells in the IPAC of the defeated mice (*P* = 0.0084, *t*(9) = 3.355; Fig. 2c,d). These findings indicate that dopamine D1 receptor in the mPFC regulates social defeat stress-induced neuronal activation positively in the IPAC.

**Figure 3:**
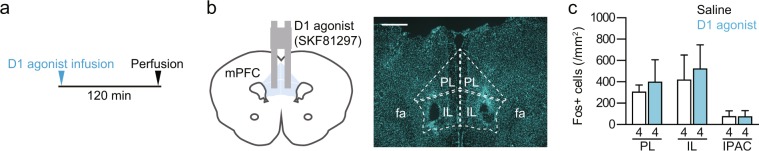
Local infusion of SKF81297, a D1-like receptor agonist, did not induce c-Fos expression in the IPAC.(**a**) An experimental schedule. Mice received bilateral infusions of a D1-like receptor agonist (SKF81297) to the mPFC. After 120 minutes, they were sacrificed for immunohistochemistry. (**b**) A representative image showing the locations of tips of cannulas in the mPFC with nuclear counterstaining by Hoechst33342. Scale bar: 200 μm. (**c**) Quantification of the numbers of Fos + cells induced by local infusion of saline or SKF81297 to the mPFC. The number of data points for each group is shown below each bar. Values are expressed as means ± SEM. Stimulation of D1-like receptors did not increase the number of c-Fos-positive cells in IPAC, IL or PL. Schematics were drawn based on the Allen Mouse Brain Atlas. See the legend of Fig. 1 for abbreviations of the names of brain areas.

To examine whether D1 activation is sufficient to induce c-Fos expression in the IPAC, we performed c-Fos immunohistochemistry in the IPAC as well as in the PL and the IL with or without mPFC infusion of SKF81297, a D1-like receptor agonist, at 400 mg/L (1.37 mM), a dose that is larger than the reported affinity (i.e. Ki~2.2 nM) (Fig. 3a,b). This stimulation of D1-like receptors in the mPFC did not alter the number of c-Fos-positive cells in the IPAC (Fig. 3c), suggesting that activation of D1-like receptors in the mPFC is not sufficient for social defeat stress-induced c-Fos expression in the IPAC. It should be noted, however, that this treatment did not increase the number of c-Fos-positive neurons in the PL or IL either (Fig. 3c), suggesting that this treatment did not increase the excitation of mPFC neurons.

### D1-expressing mPFC pyramidal neurons send direct projections to the IPAC

**Figure 4:**
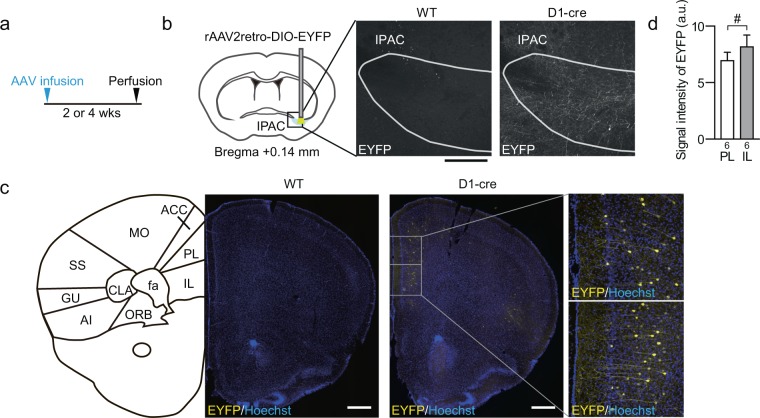
D1-expressing deep-layer pyramidal neurons in the mPFC send direct projections to the IPAC. (**a**) An experimental schedule. D1-cre mice, which express Cre recombinase in D1-expressing neurons, and wild-type mice received unilateral infusions of a retrograde AAV vector expressing EYFP in the presence of Cre recombinase (rAAV2retro-DIO-EYFP) to the IPAC. After 2 or 4 weeks, they were sacrificed for immunohistochemistry. (**b**) Representative images of EYFP expression derived from the AAV vector infused into the IPAC of D1-cre mice and wild-type mice (WT). Note that EYFP-positive projections, but not EYFP-positive cell bodies, are selectively observed in the IPAC of D1-cre mice, confirming retrograde infection of the AAV vector. Scale bar: 200 μm. (**c**) Representative images at lower and higher magnifications (middle and right, respectively) of EYFP expression (yellow) and nuclear counterstaining by Hoechst33342 (blue) in the mPFC of D1-cre mice and wild-type mice which received unilateral infusion of rAAV2retro-DIO-EYFP in the IPAC. Note that cell bodies of pyramidal neurons expressing EYFP are observed in mPFC deep layers. Scale bar: 500 μm. (d) Quantification of EYFP intensities in PL and IL in D1-cre mice which received an infusion of the AAV vector in the IPAC. The number of data points for each group is shown below each bar. The density of EYFP-expressing neurons appears to be higher in the IL than in the PL. ^#^*P* < 0.1 for paired *t* test. Schematics were drawn based on the Allen Mouse Brain Atlas. See below and the legend of Fig. 1 for abbreviations of the names of brain areas. MO; motor cortex, SS; somatosensory cortex, CLA; claustrum, GU; gustatory cortex, AI; agranular insular cortex, ORB; orbital cortex.

To examine whether the IPAC receives direct projections from D1-expressing neurons in the mPFC, we injected retrograde AAV vectors (rAAV2retro) expressing EYFP only in the presence of Cre recombinase into the IPAC of transgenic mice expressing Cre recombinase under the promoter of dopamine D1 receptor (D1-cre) or wild-type mice without Cre expression (Fig. 4a). These AAV vectors would be retrogradely infected into and consequently induce EYFP expression in D1-expressing neurons in the mPFC, if these neurons would send direct projections to the IPAC. Neither the mPFC nor the IPAC showed EYFP expression in the wild-type mice (Fig. 4b,c), confirming the lack of leaky expression of EYFP in the absence of Cre recombinase. By contrast, in D1-cre mice, EYFP expression was predominantly detected in deep-layer pyramidal neurons in the mPFC ipsilateral to the IPAC with AAV infusion. The IL tended to show higher intensity of EYFP signals than the PL (*P* = 0.0738, *t*(5) = 2.255), suggesting that mPFC neurons projecting to the IPAC are more frequently distributed in the IL (Fig. 4d). EYFP-positive neurons were also detected in the agranular insular cortex (AI; Fig. 4c), though the number was much fewer. Note that EYFP-positive axonal processes were strongly detected in the IPAC, indicating that the infected neurons send projections to the IPAC (Fig. 4b). These findings indicate that D1-expressing deep-layer pyramidal neurons in the mPFC send direct projections to the IPAC.

## Discussion

In the present study, we found that single exposure to social defeat stress induced c-Fos expression in multiple brain areas. Among these brain areas, in the NAc, LSv, BNST, IPAC, CeA, CoA and PA, social defeat stress specifically induced c-Fos expression relative to novelty-induced exploration. Furthermore, knockdown of dopamine D1 receptor in the mPFC decreased c-Fos expression in the IPAC. Using retrograde AAV vectors and transgenic mice expressing Cre recombinase under the D1 promoter, we found that D1-expressing neurons in the mPFC send direct projections to the IPAC. Thus, these findings identified distinct brain areas that are specifically activated by social defeat stress, and indicate that dopamine D1 receptor signaling in the mPFC regulates stress-induced activation of the IPAC, perhaps through direct projections.

Our findings revealed two types of brain areas with different stimulus selectivity: brain areas activated specifically by social defeat stress (stress-specific brain areas) and those activated by both social defeat stress and novelty-induced exploration (stress/exploration-responsive brain areas). The stress-specific brain areas include the extended amygdala composed of BNST, IPAC and CeA. The extended amygdala has been shown to be activated by aversive stimuli and involved in fear memory, anxiety-like behaviors and pain-induced aversion^15^. Given that social defeat stress is more aversive than novelty-induced exploration, the higher level of aversiveness could explain social defeat stress-specific activation of the extended amygdala.

The present study identified the IPAC as brain areas in which dopamine D1 receptor in the mPFC affects social defeat stress-induced c-Fos expression. Previous studies showed that systemic administration of antidepressants or antipsychotics induces Fos-like immunoreactivity in the IPAC^16,17^. Rodent studies using *in vivo* microdialysis have shown that conventional antidepressants which inhibit serotonin and/or noradrenaline reuptakes increase the extracellular level of dopamine in the mPFC^18,19^. Thus, dopamine D1 receptor in the mPFC could also be involved in antidepressant-induced Fos expression in the IPAC. However, pharmacological stimulation of D1-like receptors in the mPFC did not induce c-Fos expression in the IPAC. Since this stimulation did not induce c-Fos expression in the mPFC either, stimulation of D1 receptor alone may not be sufficient to increase the excitation of mPFC neurons. It has been reported that NMDA receptor is involved in dopamine-induced c-Fos expression in striatal neurons^20^. Thus, concurrent stimulation of dopamine receptors with NMDA receptor could be necessary to increase the excitation of mPFC neurons.

Using retrograde AAV vectors and D1-cre mice, we found that the IPAC receives direct projections from D1-expressing mPFC neurons. Given that mPFC neurons projecting to the IPAC are glutamatergic, these direct projections could facilitate social defeat stress-induced excitation of IPAC neurons. Whether direct projections from the mPFC to the IPAC are involved in antidepressant-like actions of dopamine D1 receptor in the mPFC warrants to be investigated. However, the possibility that some indirect projection mediates the effect of mPFC D1 receptor on the IPAC cannot be excluded, until the expression of dopamine D1 receptor would be manipulated selectively in mPFC neurons directly projecting to the IPAC. Since D1-expressing neurons also exist in the IPAC, the dopaminergic system may also directly affect IPAC neurons^21^.

In conclusion, the present study identified the IPAC as a brain area in which social defeat stress-induced c-Fos expression is affected by dopamine D1 receptor in the mPFC. To examine the roles of this brain area in antidepressant-like effects of dopamine D1 receptor in the mPFC may provide hints toward identifying neuronal circuits that underlie adaptive stress responses.

## Methods

### Animals

Male C57BL/6 N mice of 7 to 8-week old and male ICR mice retired from breeding were purchased from Japan SLC (Shizuoka, Japan) and used for behavioral and histological analyses. Drd1a-Cre mice (D1-cre mice, expressing Cre recombinase under the promoter of the dopamine D1 receptor) on a C57BL/6 background (B6.FVB(Cg)-Tg(Drd1a-cre)EY262Gsat/Mmucd) were provided by the Mutant Mouse Research and Rescue Center (MMRRC) at UC Davis (Davis, CA, USA) and used for histological analysis. All the mice were maintained on a 12 h light/12 h dark cycle with food and water available ad libitum. All procedures for animal care and use were in accordance with the National Institutes of Health Guide for the Care and Use of Laboratory Animals and were approved by the Animal Care and Use Committees of Kobe University Graduate School of Medicine.

### Social defeat stress

Male C57BL/6 N mice (9 to 10-week old in Fig. 1 and 14-week old in Fig. 2) were subjected to single exposure of social defeat stress, as previously described with minor modifications^9,10,22,23^. Prior to the stress, ICR mice were screened as aggressor mice based on their aggression against novel C57BL/6 N mice for 3 min daily for 3 consecutive days. The aggression was evaluated by the latency to the first attack and the frequency of the attacks during the 3-min exposure. On the day of the social defeat stress, a male C57BL/6 N mouse was transferred to the home cage of one of the aggressor mice and was attacked for 10 min. The defeated mice were sacrificed at 90 min after the stress for histological analyses.

### Immunohistochemistry

For c-Fos immunohistochemistry, we used 9 mice for the naïve group, 12 mice for the exploration group and 13 mice for the defeat stress group in Fig. 1, 5 mice for the control miRNA group and 6 mice for the D1 miRNA group in Fig. 2 (see the definitions of respective groups in the Results section), and 4 mice each for D1-like receptor agonist infusion and for saline infusion in Fig. 3. We did not exclude any of them from our analyses, unless a brain slice or its part was damaged during the immunohistochemistry procedure.

Immunohistochemistry was performed as previously described with minor modifications^10,23^. Briefly, mice were deeply anesthetized with intraperitoneal injection of sodium pentobarbital (100 mg/kg, Nacalai Tesque, Kyoto, Japan) and transcardially perfused with a flush of saline followed by 0.1 M sodium phosphate buffer containing 4% paraformaldehyde. Brains were obtained from the mice and postfixed in the same fixative at 4 °C overnight. For c-Fos immunohistochemistry, frozen sections were used. After cryoprotection in Dulbecco’s modified phosphate-buffered saline (D-PBS) containing 30% sucrose two overnights, the brains were embedded and frozen in OCT compound (Sakura Finetek, Tokyo, Japan) and cut into 30- μm sections using a cryostat (CM1860, Leica Biosystems, Wetzlar, Germany). For immunohistochemistry for anatomical tracing, the brains were cut into 50- μm sections using a vibratome (NLS-AT, Dosaka EM, Kyoto, Japan). The tissue sections were kept in D-PBS containing 25% glycerol and 25% propylene glycol at −30 °C until use. For immunohistochemistry, the sections were incubated in blocking solution (D-PBS containing 1% normal donkey serum (017-000-121, Jackson ImmunoResearch Laboratories, Inc, West Grove, PA, USA) and 0.3% Triton X-100) for 1 h at room temperature (RT), followed by incubation in the blocking solution containing primary antibodies against c-Fos derived from rabbits (sc-52; Santa Cruz Biotechnology, Santa Cruz, CA, USA; 1:500) at 4 °C for 2 days. The sections were then incubated with Alexa Fluor 555-conjugated anti-rabbit IgG antibodies (A31572; Thermo Fisher Scientific, Waltham, MA, USA; 1:1000) at 4 °C for 1 day. The sections were rinsed with D-PBS containing 0.3% Triton X-100 (PBS-T) at RT 3 times after both the primary antibody and secondary antibody reactions. After rinse in D-PBS, the sections were incubated with D-PBS containing Hoechst33342 (Thermo Fisher Scientific; 1:5000) at RT for 15 min. After wash in D-PBS at RT twice, the sections were dried on APS-coated glass slides (Matsunami Glass, Kishiwada, Japan) and mounted in the ProLong Gold antifadant (ThermoFisher Scientific). For c-Fos immunohistochemistry, fluorescent images were acquired through a 10x Plan Apo λ objective lens (N.A. 0.45) attached with a fluorescent microscope (BZ-X710, Keyence, Osaka, Japan). For immunohistochemistry for anatomical tracing, fluorescent images were acquired through a 10x Plan Fluor objective lens (N.A. 0.30) attached with a fluorescent microscope (BZ-X710, Keyence) or through a 20x Plan-APOCHROMAT objective lens (N.A. 0.8) attached with a laser-scanning confocal microscope (LSM700, Carl Zeiss Microscopy, Goettingen, Germany).

To analyze c-Fos expression, we defined brain areas to be analyzed based on the Allen Mouse Brain Atlas and a mouse brain atlas^24^, as previously described^25^. We applied the Transfluor application module of the Metamorph software (Molecular Devices Corporation, PA, USA) to detect and count objects that are within the range of 9–30 μm in diameter and brighter than a threshold determined by adjacent background signals in each brain area in each hemisphere. These objects were defined as c-Fos-positive nuclei. We averaged the numbers of c-Fos-positive cells from each hemisphere.

### Brain infusion of adeno-associated virus vectors

For knockdown of dopamine D1 receptor, adeno-associated virus (AAV) vectors expressing either artificial microRNA (miRNA) targeting dopamine D1 receptor (D1 miRNA) or negative control miRNA were produced, as previously described^10^. Briefly, these AAV vectors encode artificial microRNA targeting either dopamine D1 receptor (D1 miRNA) or control sequence (Ctrl miRNA), and emerald green fluorescent protein (EmGFP), both of which were placed in double-floxed inverse open reading frames (DIOs), thereby allowing expression of microRNAs along with EmGFP only in the presence of Cre recombinase. These AAV vectors (AAV-DIO-EmGFP-D1miRNA or AAV-DIO-EmGFP-CtrlmiRNA) were used with another AAV vector constitutively expressing Cre recombinase under the CMV promoter (AAV-Cre). For retrograde tracing experiments, an rAAV2retro vector, a recently evolved variant of AAV that infects neurons via a retrograde access, encoding enhanced yellow fluorescent protein (EYFP) inserted into a DIO cassette (rAAV2retro-DIO-EYFP) was produced, as previously described^26^.

For AAV infusion, stereotaxic surgeries were performed, as previously described with minor modifications^10^. For D1 knockdown experiments, after mice were anesthetized with isoflurane, their skulls were fixed on a stereotaxic apparatus (Stoelting, Wood Dale, IL, U.S.A). Then we vertically injected 500 nL of artificial cerebrospinal fluid (124 mM NaCl, 3 mM KCl, 26 mM NaHCO_3_, 2 mM CaCl_2_, 1 mM MgSO_4_, 1.25 mM KH_2_PO_4_, 10 mM D-Glucose) containing 5.0 × 10^8^ copies of each AAV vector into the mPFC (1.8 mm anterior, 0.4 mm lateral and 2.8 mm ventral from the bregma) of both hemispheres. Stereotaxic coordinates were determined based on a mouse brain atlas^24^. Social defeat stress was applied after 4-week recovery. For retrograde tracing experiments, after mice were anesthetized with isoflurane, their skulls were fixed on a stereotaxic apparatus. Then we vertically injected the same volume and concentration of rAAV2-retro-EYFP unilaterally into the IPAC (0.0 mm anterior, 2.2 mm lateral and 4.9 mm ventral from the bregma) of D1-cre mice or wild-type mice. Immunohistochemistry was performed after 2- or 4-week recovery.

### Infusion of a D1-like receptor agonist to the mPFC

Infusion of SKF81297, a D1-like receptor agonist, to the mPFC was performed, as described previously with minor modifications^11^. Briefly, after mice were anesthetized with isoflurane, their skulls were fixed on a stereotaxic apparatus. Then guide cannulas of 0.5-mm thickness were vertically implanted into the mPFC (1.8 mm anterior, 0.4 mm lateral and 2.8 mm ventral from the bregma) of both hemispheres and fixed with cement on the skull. The stereotaxic coordinate was determined based on a mouse brain atlas^24^. After 1-week recovery, 500 nL of SKF81297 dissolved in saline at 400 mg/L, or saline as a vehicle control was infused bilaterally into the mPFC at 100 nL/min. After 2 h, mice were deeply anesthetized with intraperitoneal injection of sodium pentobarbital and transcardially perfused with 0.1 M sodium phosphate buffer containing 4% paraformaldehyde for c-Fos immunohistochemistry.

### Statistical analyses

Data are expressed as means ± SEM. For comparisons between two groups, unpaired *t* test was used, except for Fig. 4d, in which paired *t* test was used. For comparisons between more than two groups, one-way ANOVA followed by multiple comparison tests with Tukey-Kramer correction was used. All statistical analyses were performed with Prism 8 (GraphPad Software, San Diego, CA, USA). *P* values less than 0.05 were considered to be statistically significant.

## Data Availability

The datasets generated during and/or analyzed during the current study are available from the corresponding author on reasonable request.
